# *Lactobacillus murinus* ZNL-13 Modulates Intestinal Barrier Damage and Gut Microbiota in Cyclophosphamide-Induced Immunosuppressed Mice

**DOI:** 10.3390/foods14081416

**Published:** 2025-04-19

**Authors:** Yihan Dong, Luyao Zhang, Di Qiu, Renxin Yao, Haitao Jia, Haiyang Wang, Luyao Zhou, Jiantao Zhang, Na Zhang

**Affiliations:** 1 College of Veterinary Medicine, Northeast Agricultural University, Harbin 150030, China; 18624133998@163.com (Y.D.); zhangluuuyao@163.com (L.Z.); qd13836005047@163.com (D.Q.); yrxwyyx2580@163.com (R.Y.); vetjht2022@163.com (H.J.); wanghaiyangwt@163.com (H.W.); zhouluyaoo@163.com (L.Z.); 2 Heilongjiang Provincial Key Laboratory of Pathogenic Mechanism for Animal Disease and Comparative Medicine, Harbin 150030, China; 3 Key Laboratory of Dairy Science, Ministry of Education, Northeast Agricultural University, Harbin 150030, China

**Keywords:** *Lactobacillus murinus*, functional food, immunosuppression, intestinal injury, intestinal flora

## Abstract

Cyclophosphamide (CTX) is a widely used anticancer drug in clinical practice; however, its administration can lead to gastrointestinal damage and immune suppression. *Lactobacillus murinus* (*L. murinus*) has been shown to regulate immune cell activity and protect the gastrointestinal system, showing potential application as a functional food. The objective of this study was to investigate the effects of *L. murinus* ZNL-13 on CTX-induced intestinal mucosal injury and gut microbiota in mice. The results demonstrated that *L. murinus* ZNL-13 significantly alleviated weight loss and intestinal pathological damage. Moreover, in CTX-induced intestinal injury mice, *L. murinus* ZNL-13 enhanced the release of immune factors, suppressed cell apoptosis, and protected the intestinal mucosal barrier. Additionally, it activated the TLR4/NF-κB pathway, thereby promoting immune cell activity. Furthermore, *L. murinus* ZNL-13 contributed to the restoration of gut microbial homeostasis by increasing the relative abundance of short-chain fatty acid-producing bacteria. Taken together, this investigation highlights the potential of *L. murinus* ZNL-13 in protecting the intestinal barrier and enhancing immune function while laying the groundwork for its development as a novel probiotic and functional food.

## 1. Introduction

Cyclophosphamide (CTX) is currently a widely used broad-spectrum anticancer drug in clinical practice that is commonly used to treat leukemia, lung cancer, breast cancer, and other diseases [1]. Cyclophosphamide (CTX) is a DNA-alkylating agent belonging to the nitrogen mustard class. Its anticancer activity is primarily mediated through metabolic conversion to the cytotoxic metabolite phosphoramide mustard, which disrupts DNA and protein synthesis in cells [2]. Long-term use of CTX may lead to more side effects, such as damage to the gastrointestinal mucosal barrier, immunosuppression, and acute cytotoxicity [3,4]. Additionally, CTX has been reported to induce dysbiosis of the intestinal microbiota, increasing the risk of other immune deficiency-related diseases [5]. It also promotes the colonization of harmful bacteria, disrupts intestinal barrier function, and disturbs the intestinal microbiota balance, affecting the quality of life of patients [6,7]. Therefore, regulating the gut microbiota, inflammation, and intestinal barrier may be a novel potential therapeutic strategy for treating intestinal damage. In recent years, numerous functional foods have emerged to mitigate the side effects of chemotherapy in patients, including natural plant polysaccharides, betulinic acid, probiotics, and others [8,9].

The intestinal tract, as the primary organ for digestion and nutrient absorption, also functions as a critical defense barrier [10]. Serving as a multifunctional system, it operates in close coordination with the intestinal epithelium, neuroendocrine system, innate immunity, and adaptive immunity to maintain its normal barrier function [11,12,13]. A healthy intestine can enhance digestive, absorptive, and metabolic efficiency; strengthen resistance against pathogens; and play a significant role in maintaining immune function in the body [14,15]. Simultaneously, the intestines host a diverse array of bacteria, forming a complex and dynamic ecosystem known as the gut microbiota [16]. The tight junction proteins, intestinal mucus layer, and intestinal epithelial cells collectively form the physical barrier of the intestine [17]. They protect the intestinal structure by maintaining the permeability and integrity of the intestinal mucosa, preventing intestinal bacteria, toxins, and other substances from entering other tissues [18,19]. Intestinal immunity comprises various immune cells such as T cells, B cells, innate lymphoid cells (ILCs), and macrophages [20]. Together, they regulate the production of immune cell factors and immunoglobulins, constituting a crucial defense mechanism of the body’s immune system [21,22]. Therefore, maintaining intestinal health is of paramount importance.

With their beneficial effects on host health, probiotics present a promising strategy for reducing chemotherapy-related side effects [23]. Probiotics can increase the expression of intestinal tight junctions to protect the intestinal integrity of epithelial cells in mice. In addition, they promote immune cell activity and alleviate inflammation [24]. At the same time, their massive colonization continuously consumes oxygen, creating an anaerobic environment, which deprives pathogenic bacteria of oxygen and reduces their ability to reproduce, thereby regulating the gut flora environment [25]. Previous studies have demonstrated that *Lactobacillus plantarum* has been shown to enhance intestinal barrier function and alleviate tissue damage [26]. In contrast, *Lactobacillus murinus* can increase the proportion of M2 macrophages, modulate immune cell activity, promote IL-10 release, improve immune function, and alleviate intestinal damage [27]. Furthermore, extracellular vesicles derived from *Lactobacillus murinus* can enhance immune cell activity, boost intestinal immune function, and repair the intestinal barrier in mice [28]. Additionally, feeding *Lactobacillus murinus* HF12 to neonates promotes the colonization of beneficial bacteria, reduces the abundance of pathogenic microbiota, and protects the intestine, thereby lowering the incidence of necrotizing enterocolitis [29]. Moreover, *Lactobacillus murinus* effectively alleviates *Candida albicans* intestinal infection in mice, activates AhR to regulate the Th17/Treg imbalance, reduces intestinal mucosal damage, and protects the intestinal barrier [30,31]. Toll-like receptors are key receptors for recognizing Gram-positive bacteria [27,32]. Currently, *Lactobacillus murinus* has been shown to possess immunomodulatory properties, alleviate intestinal mucosal injury, and regulate microbial homeostasis. However, its potential protective effects against CTX-induced intestinal damage remain unclear. Therefore, this study aimed to investigate the protective mechanisms of *L. murinus* ZNL-13 in a mouse model of CTX-induced immunosuppression and intestinal injury. This provides a foundational basis for its application as a novel dietary food additive to counteract immune dysfunction during tumor therapy and to serve as a daily immune modulator.

## 2. Materials and Methods

### 2.1. Bacterial Strains

*L. murinus* ZNL-13 was isolated and purified from the feces of healthy police dogs at the Heilongjiang Province Police Dog Base. The probiotic isolation method involved direct sampling [33]. *L. murinus* ZNL-13 was cultured in sterile MRS broth at 37 °C for 14 h to obtain a bacterial suspension.

### 2.2. Experimental Animals and Sample Collection

A total of 50 4-week-old Kunming mice were purchased from Liaoning Changsheng Biotechnology Co., Ltd. (Shenyang, Liaoning, China). All mice were housed under controlled conditions of temperature (23 °C) and humidity (60%), with a 12 h light–dark cycle and ad libitum access to food and water. After one week of acclimatization, the experiments were conducted. All animal studies (including the mice euthanasia procedure) were performed in compliance with the regulations and guidelines of the Northeast Agricultural University animal care and conducted according to the AAALAC and the IACUC guidelines. All experiments were approved by the Animal Welfare Committee of Northeast Agricultural University (NEAUEC2023 03 127).

**Figure 1:**
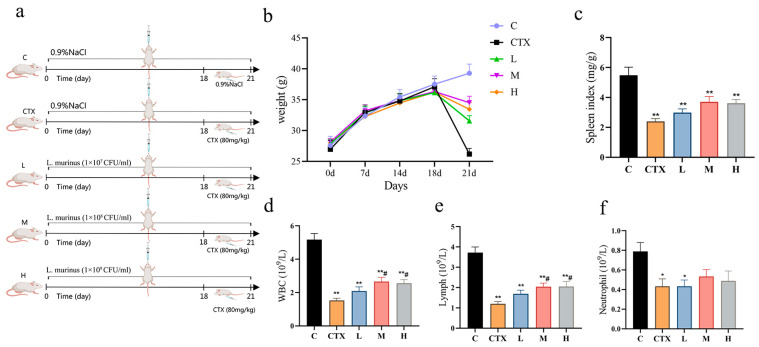
A schedule of experimental procedures (**a**); the effects of *L. murinus* ZNL-13 on the body weight (**b**); spleen index (**c**); WBCs (**d**); Lymphocytes (**e**); and Neutrophils (**f**) of immunosuppressed mice. Compared with the C group, * *p* < 0.05, ** *p* < 0.01; compared with the CTX group, ^#^ *p* < 0.05.

All mice were randomly divided into 5 groups: control group (C group), cyclophosphamide model group (CTX group), low-dose (L group), medium-dose (M group), and high-dose (H group) *L. murinus* ZNL-13 treatment groups. From days 1 to 21, mice in the control group and model group were orally gavaged with 200 μL of physiological saline daily. Mice in the low-, medium-, and high-dose groups were orally administered with fresh *L. murinus* ZNL-13 suspension at concentrations of 1 × 10^7^, 1 × 10^8^, and 1 × 10^9^ CFU/mL, respectively. Immunosuppressive model was induced by CTX according to previous study. On day 19, except for the control group, all groups were intraperitoneally injected with CTX at a dose of 80 mg/kg for 3 consecutive days [34]. Control group was administered intraperitoneally with the same volume of physiological saline. After 24 h from the last administration, all animals were fasted for 12 h, and then Isoflurane inhalation anesthesia was used to collect blood from the retro-orbital venous plexus, followed by cervical dislocation to euthanize the mice. Colon, spleen, and feces were then collected (Figure 1a). Fresh blood samples were collected in additive-free blood collection tubes, while feces and fresh tissue samples were placed in sterile cryogenic tubes and rapidly frozen in liquid nitrogen before transfer to −80 °C.

### 2.3. Body Weight and Immune Organ Weight Analysis

The weight of the mice was recorded. After euthanasia, the weight of the spleen was measured. The spleen and thymus index were calculated according to the following formula: spleen and thymus index  =  spleen and thymus weight (mg)/body weight (g) [35].

### 2.4. Blood Cell and Serological Analysis

Blood was collected using EDTA-K2 anticoagulation tubes, and the amounts of white blood cells (WBC), lymphocytes (Lymph), and neutrophils (Neut) were assessed using a fully automated hematology analyzer (Mairui, Shenzhen, China).

According to the manufacturer’s protocol use, an assay kit was used to detect the levels of IgG, MDA, GSH, SOD, DAO, D-LAC, IL-1ß, IL-6, TNF-α, IL-2, and IL-10 in the serum and intestinal tissues (Jingmei Biological Technology, Yancheng, China). In addition, a high-speed homogenizer was used to homogenize a mixture of intestinal tissue and sterile saline. The supernatant was collected after centrifugation at 10,000× *g* for 10 min at 4 °C.

### 2.5. Histological Analysis

The colon samples were treated with a 4% paraformaldehyde solution to fix them and then embedded in paraffin. Next, the samples were sectioned into 4 μm slices and stained using hematoxylin and eosin. Finally, the samples were viewed under light microscopy (BX-FM; Olympus Corp., Tokyo, Japan) at a magnification of 200×.

### 2.6. mRNA Detection in the Intestine

Total RNA from mouse intestinal tissue was extracted using the Total RNA Extraction Kit (Aidlab, Beijing, China). The extracted RNA was subjected to genomic DNA removal and reverse transcription reaction using a reagent kit (Takara, Dalian, China). For qPCR amplification, TB Green^®^ Premix Ex Taq™ II FAST qPCR was used with the addition of TB Green Premix Ex Taq II FAST qPCR (2×), ddH_2_O, and forward and reverse primers for amplification (Table 1). The fluorescence intensity was monitored throughout the PCR process using the Light Cycler^®^480 from Roche (Roche, Germany), and cycle threshold (Ct) values were obtained. The gene relative abundance of mRNA was calculated with the 2^−ΔΔCt^ method, and β-actin was an internal reference gene.

### 2.7. Western Blot Analysis

The total protein from intestinal tissue was extracted using the traditional protein extraction method with RIPA lysis buffer. The total protein concentration and purity of the samples were determined using a BCA reagent kit. Protein content analysis was performed using SDS-PAGE and Western blotting with NC membranes. After electrophoresis, the membranes were blocked with 5% skim milk powder at 4 °C overnight and then incubated with primary antibodies: β-actin (1:5000, Bioss, Beijing, China), p65 (1:3000, Proteintech, Wuhan, China), TLR4 (1:4000, Proteintech), MYD88 (1:1500, Wanlei, Shenyang, China), Claudin-1 (1:1500, Wanlei, China), Occludin (1:1000, Wanlei, China), ZO-1 (1:1000, Wanlei, China), Bcl-2 (1:1000, Affinity, Liyang, China), Bax (1:4000, Proteintech), caspase-3 (1:1000, Wanlei, China), caspase-8 (1:1000, Wanlei, China), caspase-9 (1:1000, Wanlei, China), and FAS (1:1000, Wanlei, China). After incubation, the membranes were washed three times with TBST and then incubated with secondary antibodies at room temperature for 1.5 h, followed by washing. Protein signals were detected using an ultra-sensitive ECL reagent kit, and images were captured and analyzed using ImageJ (1.53c).

### 2.8. 16S rRNA Intestinal Content Microbiota Analysis

Microbial community total DNA was extracted according to the instructions of the E.Z.N.A.^®^ DNA kit (Omega Bio-tek, Norcross, GA, USA). PCR amplification of the V3–V4 region of the 16S rRNA gene was performed using upstream primers 338F (5′-ACTCCTACGGGAGGCAGCAG-3′) and 806R (5′-GGACTACHVGGGTWTCTAAT-3′). Purified amplicons were pooled in equimolar amounts and paired-end sequenced on an Illumina PE300 platform (Illumina, San Diego, CA, USA) according to the standard protocols by Majorbio Bio-Pharm Technology Co., Ltd. (Shanghai, China). Subsequently, UPARSE v7.1 software was utilized to cluster operational taxonomic units (OTUs) from the quality-controlled assembled sequences at a 97% similarity threshold and to remove chimeras. The community composition of each sample was then determined at different taxonomic levels. Based on sequenced reads and OTU levels, we analyzed the microbial diversity in mice feces.

### 2.9. Statistical Analysis

GraphPad Prism 9.5 (GraphPad Software, Inc., San Diego, CA, USA) was used to analyze the data. The overall significant difference was evaluated by single-factor analysis of variance (ANOVA) and Tukey multiple comparisons. A *p* value < 0.05 was considered to be statistically significant.

### 2.10. ARRIVE Guidelines

All the research methods contained in the manuscript were carried out in accordance with the requirements of ARRIVE.

## 3. Result

### 3.1. L. murinus ZNL-13 Enhances the Growth Performance and Production of Immune Cells in CTX Mice

In the initial stage of model establishment, the five groups of mice showed similar trends in body weight growth. After intraperitoneal injection of CTX, mice in the CTX group showed rapid weight loss and significantly reduced spleen index. Additionally, compared with the C group, the CTX group exhibited messy, dull fur, depressive behavior, and decreased food intake, indicating successful modeling (Figure 1b,c). In contrast, the body weight of the L, M, and H intervention groups was significantly higher than that of the CTX group, and they all had a mitigating effect on the decrease in spleen index. This suggests that *L. murinus* ZNL-13 exerts a regulatory effect on CTX-induced immune organ atrophy, thereby enhancing immune function in mice.

The results of the blood routine analysis showed that after administration of CTX, the WBC, Lymph, and neutrophil counts in the CTX group showed a marked decreasing trend (*p* < 0.05). The L, M, and H intervention groups exhibited varying degrees of mitigation. Notably, the mitigation effects of the M and H groups were significantly better than those of the CTX group in terms of WBC and Lymph (*p* < 0.05) (Figure 1d–f).

### 3.2. L. murinus ZNL-13 Alleviates CTX-Induced Oxidative Stress, Immune-Related Cytokines, and Enhances Intestinal Function

**Figure 2:**
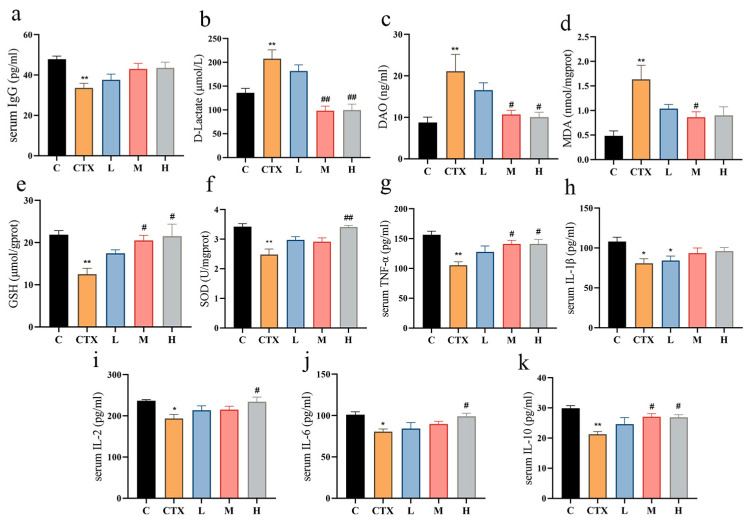
*L. murinus* ZNL-13 alleviated CTX-induced immunosuppression and oxidative stress. Immunoglobulin G (IgG) (**a**); D-Lactate (**b**); DAO (**c**); MDA (**d**); GSH (**e**); SOD (**f**); TNF-α (**g**); IL-1β (**h**); IL-2 (**i**); IL-6 (**j**); and IL-10 (**k**). Compared with the C group, * *p* < 0.05, ** *p* < 0.01; compared with the CTX group, ^#^ *p* < 0.05, ^##^ *p* < 0.01.

After further research, the influence of *L. murinus* ZNL-13 on serum IgG, intestinal functional markers, oxidative factors, and immune-related cytokines was investigated. The results indicated that compared with the C group, serum immunoglobulin IgG was significantly decreased in the CTX group, while there was an improvement in the L, M, and H groups (Figure 2a). The intestinal functional markers DAO and D-LAC were significantly increased in the CTX group. Pretreatment with *L. murinus* ZNL-13 significantly attenuated the CTX-induced elevations in serum DAO and D-LAC levels, with marked improvements observed in the M and H groups (*p* < 0.05), thereby improving intestinal function (Figure 2b,c). Meanwhile, pretreatment with *L. murinus* ZNL-13 effectively alleviated serum lipid peroxidation (Figure 2d) and enhanced antioxidant capacity (Figure 2e,f). Additionally, compared with the C group, immune-related cytokines in the intestine were significantly decreased in the CTX group. The *L. murinus* ZNL-13 intervention group mice exhibited some degree of alleviation. For TNF-α, IL-2, IL-6, and IL-10, the H group showed a significant increasing trend in the reduction in cytokines induced by CTX (*p* < 0.05) (Figure 2g–k).

### 3.3. L. murinus ZNL-13 Alleviates the Damage to Colonic Tissues Induced by CTX

**Figure 3:**
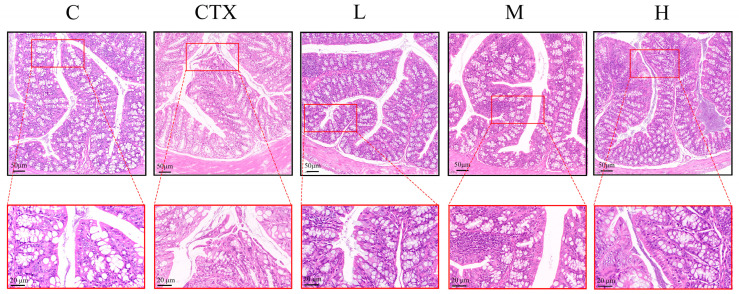
The pathological examination of intestinal tissue histology was conducted through H&E staining. Representative microphotographs were obtained at magnifications of 200× and 400×.

Colonic histopathological examination with H&E staining revealed that *L. murinus* ZNL-13 has a certain alleviating effect on intestinal damage. In the C group, the villi were intact, and the epithelial absorptive cells were tightly arranged. In the CTX group, the villi were dissolved, goblet cells were ruptured, edges were incomplete, and epithelial absorptive cells were disordered, with infiltration of inflammatory cells and an increase in the thickness of the crypt base. The groups pretreated with *L. murinus* ZNL-13 exhibited varying degrees of histological improvement characterized by intact goblet cells and well-organized glands arranged in a fence-like structure. Additionally, infiltration of inflammatory cells was observed in the lamina propria of the colon in the CTX group, which was alleviated by treatment with *L. murinus* ZNL-13 (Figure 3).

**Figure 4:**
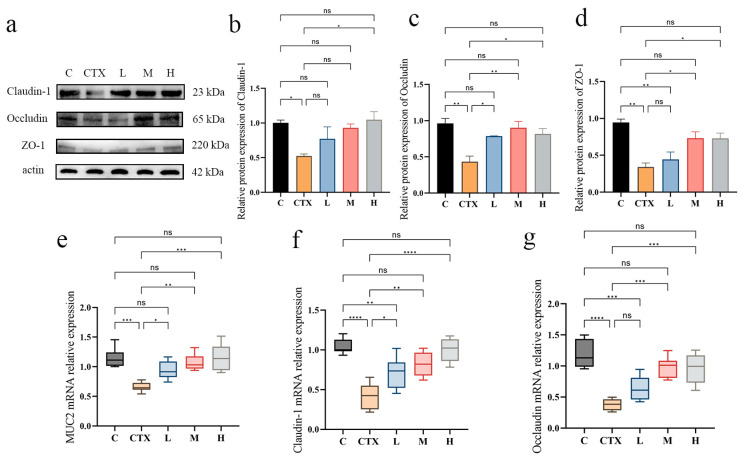
Effects of *L. murinus* ZNL-13 on the intestinal barrier pathway in the colon of immunosuppressed mice. Detection of Claudin-1, Occludin, and ZO-1 protein expression in colon tissue by Western blot (**a**); the ratios of the Claudin-1/β-actin (**b**), Occludin/β-actin (**c**), and ZO-1/β-actin (**d**) protein bands for each region were quantified using densitometry and presented in the graph. The relative mRNA expression levels of mucin MUC2 (**e**) and tight junction protein Claudin-1 (**f**), Occludin (**g**), and in the intestinal tissue. ^ns^ *p* > 0.05, * *p* < 0.05, ** *p* < 0.01, *** *p <* 0.001, and **** *p* < 0.0001.

The subsequent Western blot analysis and Q-PCR were performed to further investigate the levels of intestinal barrier-related genes (Figure 4a–g). As shown in Figure 4, compared with the blank group, the induction of CTX significantly decreased the protein levels of ZO-1, Occludin, and Claudin-1 (all *p* <  0.05). Pretreatment with *L. murinus* ZNL-13 mitigated this trend, with the H group exhibiting a significant alleviation across all three indicators (*p* < 0.05). Similarly, the RNA results followed the same pattern. In summary, these results suggest that the prophylactic use of *L. murinus* ZNL-13 can enhance the expression of the intestinal mucin protein MUC2 and tight-junction-related genes and proteins, thereby reducing intestinal permeability and strengthening intestinal barrier function. Notably, the improvement observed in the H group was more pronounced than in the L and M groups.

### 3.4. Effects of L. murinus ZNL-13 on Key Proteins in Colon of CTX Mice

**Figure 5:**
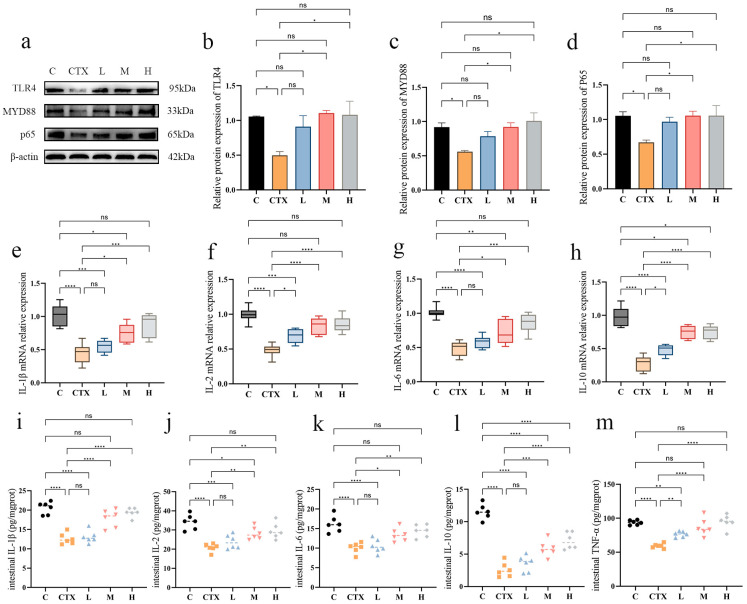
Effects of *L. murinus* ZNL-13 on TLR4/MYD88 pathway in colon of immunosuppressed mice. The protein expression of TLR4, NF-κB, and MyD88 in the colon were detected by Western blot (**a**). The ratios of TLR4/β-actin (**b**), MyD88/β-actin (**c**), and NF-κB/β-actin (**d**) protein bands for each region were quantified using densitometry and presented in the graph. The changes in mRNA levels of IL-1β, IL-2, IL-6, and IL-10 (**e**–**h**) in colonic tissues, as well as the content of TNF-α, IL-1β, IL-2, IL-6, and IL-10 in colonic tissues, were detected by ELISA (**i**–**m**). ^ns^ *p* >0.05, * *p* < 0.05, ** *p* < 0.01, *** *p* < 0.001, and **** *p* < 0.0001.

Western blot analysis indicated that the expression levels of TLR4, MYD88, and p65 proteins in the CTX group were significantly decreased compared with the control group (*p* < 0.05) (Figure 5a–d). The protein levels in the *L. murinus* ZNL-13 intervention group mice showed an upward trend, with the M and H groups displaying a significant increase compared with the CTX group (*p* < 0.05). In addition, we utilized Q-PCR (Figure 5e–h) and ELISA assay kits (Figure 5i–m) to measure the levels of intestinal immune-related factors. The results indicated that CTX significantly reduced the levels of IL-1β, IL-2, IL-6, IL-10, and TNF-α (*p* < 0.01). The expression levels of the groups pretreated with *L. murinus* ZNL-13 were all increased, with the M and H groups significantly higher than the CTX group (*p* < 0.05 and *p* < 0.01, respectively).

### 3.5. L. murinus ZNL-13 Effectively Attenuates CTX-Induced Apoptosis in Intestinal Tissue Cells

**Figure 6:**
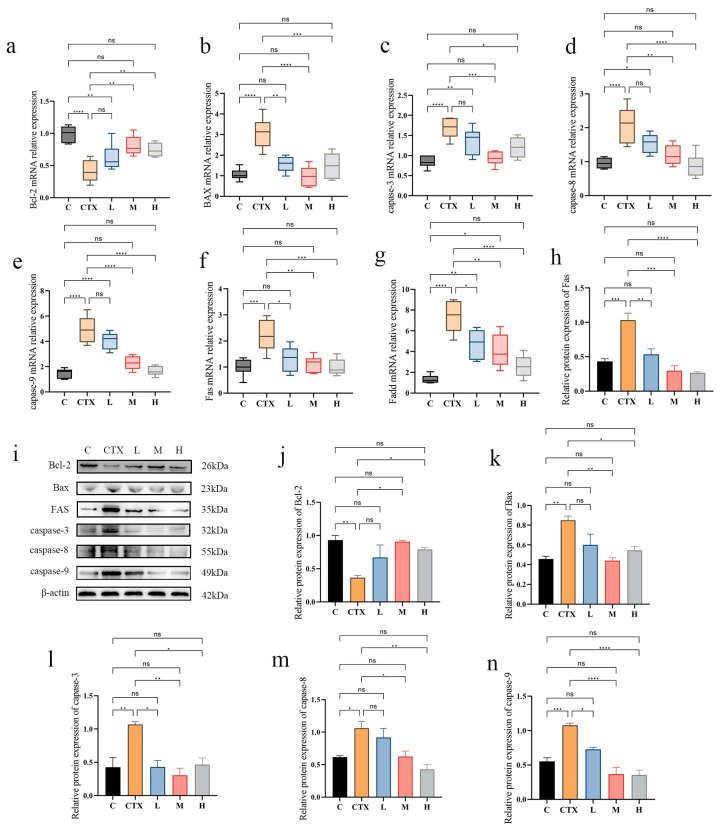
Effects of *L. murinus* ZNL-13 on the apoptosis pathway in the colon of immunosuppressed mice. The expression levels of mRNA for Bcl-2 (**a**), Bax (**b**), caspase-3 (**c**), caspase-8 (**d**), caspase-9 (**e**), FAS (**f**), and FAdd (**g**). The protein expression of Bcl-2, Bax, FAS, caspase-3, caspase-8, and caspase-9 in the colon were detected by Western blot (**i**). The ratios of Fas/β-actin (**h**), Bcl-2/β-actin (**j**), Bax/β-actin (**k**), caspase-3/β-actin (**l**), caspase-8/β-actin (**m**), and caspase-9/β-actin (**n**) protein bands for each region were quantified using densitometry and presented in the graph. ^ns^ *p* > 0.05, * *p* < 0.05, ** *p* < 0.01, *** *p* < 0.001, and **** *p* < 0.0001.

CTX can induce both intrinsic and extrinsic apoptosis. To validate the mechanism by which *L. murinus* ZNL-13 mitigates apoptosis, further investigation into apoptosis-related genes and proteins was conducted (Figure 6). At the mRNA expression level, apoptosis-related genes including Bax, FAS, FADD, caspase-3, caspase-8, and caspase-9 show an upregulation trend in the CTX group. Conversely, the trend is opposite in the L, M, and H groups, with significant differences observed in the M and H groups (*p* < 0.05 and *p* < 0.01). Additionally, Bcl-2 is downregulated in the CTX group but significantly alleviated in the M and H groups (*p* < 0.01). Further Western blot analysis confirmed these results, with significant upregulation of apoptosis-related genes in the CTX group and significant alleviation in the M and H groups. These results indicate that pretreatment with *L. murinus* ZNL-13 can significantly alleviate the trend of intestinal cell apoptosis induced by CTX.

### 3.6. The Effect of L. murinus ZNL-13 on the Intestinal Microbiota

**Figure 7:**
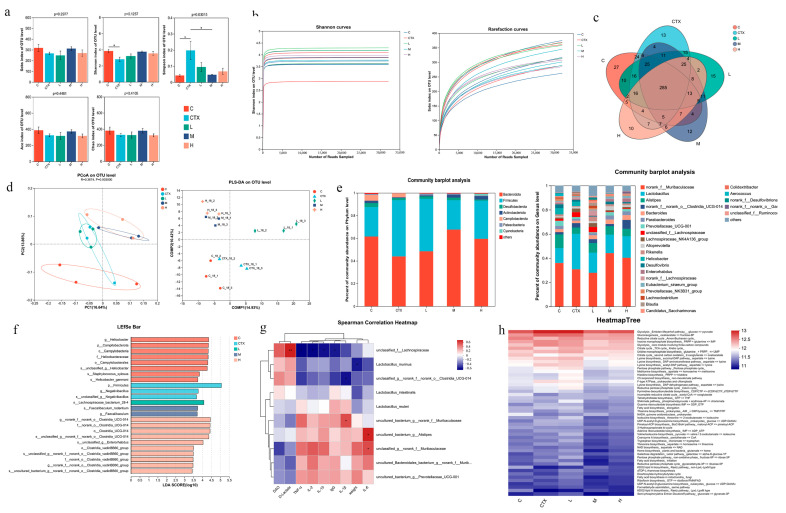
Effect of *L. murinus* ZNL-13 on gut microbiota composition Alpha diversity analysis (**a**); Shannon and sobs Rarefaction curve (**b**); Venn diagram analysis (**c**); PCoA and PLS-DA analysis (**d**); taxonomic composition distribution at the phylum and genus levels of gut microbiota in the C, CTX, L, M and H groups (**e**); analysis of differences in the microbial taxa shown by LEfSe linear discriminant analysis (LDA) coupled with effect size measurements (**f**); the Spearman’s correlation between gut microbiota and immune factors (**g**); microbial function prediction of the five groups by PICRUSt (**h**). * *p* < 0.05, ** *p* < 0.01 indicate the significant associations.

Using 16S rRNA high-throughput sequencing, we explored the effects of CTX and *L. murinus* ZNL-13 on the intestinal microbiota. Microbial diversity analysis showed the indexes of Simpson in the *L. murinus* ZNL-13 group were lower than that in the CTX group; thus, the indexes of Sobs, Chao1, Shannon, and Ace in the *L. murinus* ZNL-13 group were higher than those in the CTX group, which suggested that the intervention of *L. murinus* ZNL-13 increased the richness and diversity of the gut microbiota (Figure 7a). The Sobs and Shannon dilution curves tended to flatten (Figure 7b). The Venn diagram reflected the unique and common OTUs in the microbial composition of the five groups, with the number of shared OTUs reaching 47.19% (Figure 7c). Principal Coordinate Analysis (PCOA) and Partial Least Squares–Discriminant Analysis (PLS-DA) were used for beta diversity analysis of the community samples in each group. The results both showed significant differences between the C group and the CTX group, with the M and H groups being closer to each other and distant from the CTX group (Figure 7d). These results indicate that CTX alters the microbial community structure of the C group, and the microbial composition structures of the M and H groups are similar and significantly different from the CTX group.

To further evaluate the specific changes in gut microbiota, we investigated the differences in microbial community composition at the phylum and genus levels (Figure 7e). *Bacteroidota* and *Firmicutes* were the predominant phyla in all groups. After CTX administration, there was an increase in *Firmicutes* abundance and a decrease in *Bacteroidota* at the phylum level. In contrast, the *L. murinus* ZNL-13 pretreated group exhibited the opposite trend compared with the CTX group, with an increase in *Bacteroidota* and *Actinobacteriota* and a decrease in *Campilobacterota*, while the abundance of *Cyanobacteria* increased in the H group. At the genus level, the relative abundance of *norank_f__Muribaculaceae*, *Lachnospiraceae_NK4A136_group*, *Alistipe, Parabacteroides*, *Bacteroides*, and *Prevotellaceae_*UCG-001 in the CTX group showed a decreasing trend, while the abundance of *Lactobacillus* and *unclassified_f__Lachnospiraceae* significantly increased. *L. murinus* ZNL-13 pretreatment reversed this trend. The representative species of intestinal microbiota in each group was determined by LEfSe analysis in which the significant differences in bacterial abundance were determined by the non-parametric factorial Kruskal–Wallis sum-rank test and linear discriminant analysis (LDA) (Figure 7f). Based on LEfSe analysis, in the CTX group, the predominant microbial taxa were from the phylum *Firmicutes* and *g__Negativibacillus*. *L. murinus* ZNL-13 pretreatment altered the dominant microbial composition in the intestinal contents of the CTX-treated mice. Spearman correlation analysis was applied to assess the relationships between the intestinal microbial communities in each group and immune parameters. The top ten species ranked by correlation, D-Lactate and *unclassified_f_Lachnospiraceae*, intestinal tissue IL-1β and *uncultured_bacterium_g_norank_f_Muribaculaceae*, intestinal tissue IL-6 and *uncultured_bacterium_g_Alistipes*, *unclassified_g_norank_f_Muribaculaceae* showed a significant positive correlation horizontally (*p* < 0.05 and *p* < 0.01) (Figure 7g). PICRUSt was used for predicting changes in intergroup microbial taxonomic composition and functional annotation (Figure 7h). The heatmap illustrates enrichment of abundance in the Glycolysis and Gluconeogenesis metabolic pathways in the CTX group. In contrast, the dominant functions in the H group are the Reductive Citrate cycle and the Citrate cycle. These results further reveal that the intervention of *L. murinus* ZNL-13 could increase the immunity of mice by increasing the abundance of probiotics.

## 4. Discussion

Studies have indicated that CTX can suppress cellular and humoral immune responses. It is considered a primary choice for anticancer treatments, autoimmune diseases, and immunosuppressive regimens for organ transplantation. However, the intestinal tract epithelial barrier disruption and immune dysfunction caused by CTX can lead to opportunistic and pathogenic microbial infections [35,36,37]. The spleen index is a primary indicator for assessing immune function in the human body [6]. After intraperitoneal injection of CTX, the mice exhibited rapid weight loss, noticeable reduction in spleen index, disheveled fur, depressive behavior, and decreased food intake, suggesting successful modeling. Previous studies have shown that long-term consumption of *Lactobacillus* significantly improves immune organ indices, which aligns with the results of our study [38]. Additionally, CTX induces the generation of reactive oxygen species (ROS), leading to oxidative stress, lipid peroxidation, and cellular damage [39]. Our results indicate that prophylactic administration of *L. murinus* ZNL-13 significantly alleviates oxidative stress in the intestines and effectively mitigates oxidative damage.

Reports suggest that CTX inhibits the synthesis of tight junction proteins, increases intestinal permeability, and disrupts the intestinal mucosal barrier [8,40]. The disruption of the intestinal mucosal barrier function is a key factor in inducing intestinal damage [41]. In this study, hematoxylin and eosin (H&E) staining results revealed that the colonic wall in the CTX group exhibited thinning, inflammatory cell infiltration, villus breakage, and severe damage to goblet cells. When intestinal mucosal cells undergo necrosis and shed into the lumen and bloodstream, circulating DAO and D-Lactate levels increase. However, *L. murinus* ZNL-13 significantly alleviated this condition. Dysfunction of tight junction proteins significantly alters cellular permeability, allowing the invasion of harmful substances such as bacterial toxins, which serves as a key pathogenic mechanism in many intestinal diseases [42]. In this study, *L. murinus* ZNL-13 significantly increased the expression of Claudin-1, Occludin, ZO-1, and MUC2. Therefore, we speculate that prophylactic consumption of *L. murinus* ZNL-13 holds the potential to strengthen the intestinal barrier and repair intestinal mucosal injury by activating the expression of the intestinal tight junction protein.

Research indicates that approximately 70–80% of the body’s immune cells are aggregated within intestinal tissues [43]. Cytokines are primarily produced by macrophages and lymphocytes, both of which play important roles in immune regulation and inflammatory responses [33,44,45,46,47]. The levels of cytokines TNF-α, IL-1β, IL-2, IL-6, IL-10, and IgG in peripheral circulation and colonic tissues were significantly downregulated following CTX administration, highlighting its profound immunosuppressive effects. *L. murinus* has been reported to enhance T-cell activity, promote macrophage polarization, and stimulate the production of immune cytokines [28,48,49]. In this study, *L. murinus* ZNL-13 significantly improved this outcome. Consistent with previous findings, *Lactobacillus plantarum* KLDS1.0318 can significantly restore cytokine levels, such as IL-6 and TNF-α, reduced by CTX [50]. Multiple studies indicate that the NF-κB signaling pathway plays a crucial role in the induction and secretion of cytokine expression [34]. Additionally, impaired MyD88/p65 signaling in the intestinal epithelium is associated with the reduction in tight junction proteins such as Claudin-1 and Occludin as well as the downregulation of MUC2 expression [51,52,53]. *L. casei* NCU011054 significantly upregulated the mRNA levels of TLR2, TLR4, TLR6, p65, NF-κB, T-bet, and GATA-3; restored the number of CD4⁺ T cells; and alleviated the inhibitory effects of CTX [54]. The results showed that the NF-κB signaling pathway was significantly suppressed by CTX, while the expression of proteins related to this pathway was markedly upregulated in the *L. murinus* ZNL-13 pretreatment group. Therefore, we speculate that the mechanism by which *L. murinus* ZNL-13 regulates cytokine expression involves the activation of the TLR4 receptor, modulation of downstream NF-κB pathway expression, and ultimately, the stimulation of immune cytokine secretion and transcription factor expression, thereby restoring immune balance in CTX-induced intestinal injury in mice. However, previous studies have indicated that probiotics can also alleviate intestinal inflammation by modulating additional signaling pathways, such as MAPK, Nrf2, and PI3K/AKT [55,56,57]. Therefore, TLR4 may not be the sole receptor involved in this process. Further investigations are warranted to explore other molecular mechanisms beyond the TLR4/NF-κB axis that may contribute to *L. murinus* ZNL-13-mediated immune regulation.

CTX inhibits cellular DNA replication and differentiation; however, it also exhibits cytotoxic effects, accelerating the apoptosis of rapidly proliferating cells in the body [58,59,60]. Studies have shown that acetaminophen can promote the translocation of the pro-apoptotic factor Bax to the mitochondria and facilitate the maturation of caspase-3, ultimately leading to tissue cell damage [61]. Additionally, the inhibition of the MAPK and NF-κB signaling pathways can also trigger apoptosis [62,63]. *Lactobacillus plantarum* Lp2 effectively regulates the MAPK signaling pathway and downregulates the expression of downstream pro-apoptotic proteins, including caspase-3 and Bax, thereby attenuating CTX-induced hepatocyte apoptosis [55]. This finding indirectly suggests that the TLR4/NF-κB pathway may not be the sole mechanism through which Lactobacillus species, including *L. murinus*, exert their protective effects. Consistent with previous studies, CTX induces apoptosis, while *L. murinus* ZNL-13 upregulates Bcl-2 and downregulates the mRNA and protein expressions of Bax, Fas, caspase-3, caspase-8, and caspase-9; preserves mitochondrial membrane integrity; and blocks apoptotic signal transduction mediated by death receptor–ligand interactions, thereby mitigating excessive apoptosis in intestinal cells. These findings suggest that *L. murinus* ZNL-13 alleviates CTX-induced apoptosis and intestinal injury by suppressing apoptotic pathway activation.

The gut microbiota is a complex system influenced by factors such as the environment and diet, which can impact the composition of microbial communities [64,65]. CTX-induced immune suppression and intestinal damage disrupt the gut microbiota, the organism experiences secondary declines in immune function, which induces increased inflammation in the intestine, leading to a vicious cycle [66,67,68]. CTX has been shown to increase the Simpson index and reduce microbial diversity [69]. Similarly, our study found that prophylactic administration of *L. murinus* ZNL-13 reduced the Simpson index, which may have a beneficial impact. Both PCA and Partial Least Squares–Discriminant Analysis (PLS-DA) results indicate significant differences in microbial structures between the C group and the CTX group. *L. murinus* ZNL-13 altered the intestinal microbiota structure of mice. At the phylum level, the relative abundance of *Firmicutes* increased, while *Bacteroidetes*, *Desulfobacterota*, and *Actinobacteriota* decreased in the CTX group. *Bacteroidetes* can ferment indigestible polysaccharides to provide an energy source for the host [70]. Prophylactic administration of *L. murinus* ZNL-13 led to an increase in *Bacteroidetes*, which may enhance the digestion and absorption of polysaccharides in the intestine. *Campylobacterota* are pathogens, including members such as *Helicobacter* and *Desulfovibrio*, whose abundance increase can exacerbate inflammatory diseases [71]. A significant enrichment of *Campylobacterota* in the C and CTX groups was observed in this study, while the *L. murinus* ZNL-13 groups showed minimal presence of such microorganisms. This supports their role in promoting intestinal health, consistent with previous reports [35]. This evidence suggests that *L. murinus* ZNL-13 can reduce the activity of such microorganisms, helping the host organism restore gut homeostasis and create a healthy intestinal environment.

At the genus level, *Helicobacter,* which are highly relevant in gut microbial dysbiosis and disease, appeared in both the C and CTX groups, while they were scarcely observed in the *L. murinus* ZNL-13 groups. SCFAs provide the necessary nutrients and energy for intestinal epithelial cells, preserving the integrity and functionality of the intestinal mucosal barrier, which can block the transport of toxins between the intestine and bloodstream, inhibiting inflammation [72,73]. *Muribaculaceae*, *Bacteroides*, and *Alloprevotella* have been shown to promote the production of short-chain fatty acids (SCFAs). And these bacteria were prominently present in the *L. murinus* ZNL-13 group [74]. *Alistipes* and *Prevotellaceae_UCG-001* can produce polysaccharide-digesting enzymes that promote the production of SCFAs [75,76,77,78,79,80]. The increased relative abundance of *Alistipes* and *Prevotellaceae_UCG-001* in the M and H groups suggests that supplementing with *L. murinus* ZNL-13 promotes aiding in the restoration of gut health and enhancing food conversion and absorption capacity after CTX administration. This also helps confirm that the *L. murinus* ZNL-13 group can alleviate the rapid weight loss induced by CTX.

Additionally, differences in microbiota among groups and representative species, as well as dominant microbial communities, can be identified through LDA score histograms. *Negativibacillus,* a signature species identified in the CTX group, has been confirmed to be significantly increased in the gut microbiota of patients with ulcerative colitis [81]. However, the signature species identified in the *L. murinus* ZNL-13 intervention groups have all been reported to exert regulatory effects that contribute to host health. *Faecalibaculum rodentium* plays a beneficial role in regulating intestinal immunity and inflammation, and its independent administration has been shown to counteract the progression of colitis [82]. Furthermore, closely related genera within the *Faecalibaculum* group have been demonstrated to influence bile acid metabolism through dehydroxylation processes [83]. An increased abundance of *Clostridia UCG-014* has been associated with enhanced production of short-chain fatty acids (SCFAs) [84,85]. Notably, the *Clostridia_vadinBB60_group* has been identified as a known producer of acetate and propionate [86,87,88]. Acetic acid can enhance the production of reactive oxygen species and phagocytic activity, induce apoptosis, and modulate the recruitment of immune cells such as neutrophils [89]. Butyrate salts have been shown to enhance the activity of natural killer (NK) cells and have the function of regulating the activity of immune cells and altering the production of inflammatory cytokines [90,91]. In addition, *Clostridium* has been reported to promote the conversion of primary bile acids into secondary bile acids via the activity of 7α-dehydroxylase. Secondary bile acids have been shown to activate intestinal macrophages and Treg cells through TGR5, thereby enhancing immune responses against pathogens [92,93]. *Clostridium* also facilitates the metabolism of tryptophan into indolepropionic acid, a compound known for its antioxidant properties. Certain tryptophan metabolites have been shown to activate B cells, further contributing to immune modulation. [94,95]. Moreover, *Clostridium* species can enhance arginine metabolism, resulting in the production of polyamines such as putrescine and spermidine, which are essential for intestinal barrier repair. Notably, spermidine has been shown to enhance immune function by promoting mitochondrial metabolism in natural killer (NK) cells [96,97]. Therefore, we hypothesize that *L. murinus* ZNL-13 supplementation may improve immune function by modulating the gut microbiota, increasing the production of beneficial metabolites, enhancing intestinal barrier integrity, and promoting immune cell activity. A recent study found that *Faecalibaculum rodentium* exerts anti-tumor effects in a mouse model of colorectal cancer by producing short-chain fatty acids [98]. This also provides insight into the potential for *L. murinus* ZNL-13 to co-suppress tumor cell proliferation with CTX on the basis of alleviating CTX-induced damage. In summary, prophylactic administration of *L. murinus* ZNL-13 promotes the colonization of beneficial bacteria and restores the imbalance in the intestinal microbiota induced by CTX in mice, which is crucial for maintaining the stability of the host microbiota. It also activates immune cell activity and enhances immune function, suggesting the potential beneficial effects of *L. murinus* ZNL-13 on the intestinal microenvironment of animals or humans in the future.

In summary, prophylactic administration of *L. murinus* ZNL-13 effectively mitigated intestinal damage in CTX-treated mice to varying degrees. It significantly promoted the secretion of intestinal immune factors such as IL-1β and IL-2, activated the NF-κB pathway to stimulate immune cell activity, and restored immune function. Moreover, it downregulated apoptosis-related genes such as BAX and caspase-3 and alleviated intestinal oxidative damage, contributing to the repair of intestinal injury. Meanwhile, *L. murinus* ZNL-13 helped regulate gut microbiota homeostasis, increased the abundance of beneficial bacteria, and promoted the restoration of overall health. These findings provide a theoretical basis for the development of *L. murinus* ZNL-13 as a dietary nutritional supplement.

## 5. Conclusions

This study investigated the potential protective mechanisms of *L. murinus* ZNL-13 against CTX-induced immunosuppression and intestinal injury in mice and further revealed the possible role of the gut microbiota in alleviating intestinal damage and activating immune function. These findings support the potential application of *L. murinus* ZNL-13 as a novel functional food additive for humans or other animals.

## Figures and Tables

**Table 1:** Primer sequence.

Target Gene	Forward	Reverse
β-actin	GATATCGCTGCGCTGGTCG	CATTCCCACCATCACACCCT
Claudin1	AAAGCACCGGGCAGATACAG	TCATGCCAATGGTGGACACA
Occludin	TCCACCTCCTTACAGACCTGA	AAGAGTACGCTGGCTGAGAG
MUC2	TCCTGACCAAGAGCGAACAC	ACAGCACGACAGTCTTCAGG
IL-1β	AATGCCACCTTTTGACAGTGAT	ATCAGGACAGCCCAGGTCAA
IL-2	GCGGCATGTTCTGGATTTGAC	CCTCAGAAAGTCCACCACAGT
IL-6	TTCCTCTGGTCTTCTGGAGT	TCTGTGACTCCAGCTTATCTCTTG
IL-10	CCTGGGTGAGAAGCTGAAGAC	CTTGTAGACACCTTGGTCTTGG
TNF-α	CCCTCACACTCACAAACCAC	ACAAGGTACAACCCATCGGC
Bcl-2	CTTTGAGTTCGGTGGGGTCAT	GCCAGGAGAAATCAAACAGAGG
Bax	CACTAAAGTGCCCGAGCTGA	GGAGAGGAGGCCTTCCCAG
FAS	TGCTTGCTGGCTCACAGTTAAG	GAACCCGCCTCCTCAGCTT
FADD	GTGTGTGACAATGTGGGGAG	GACTCTCCCTTACCCGCTCA
Caspase-3	ATGGGAGCAAGTCAGTGGAC	GTCCACATCCGTACCAGAGC
Caspase-8	CGGGAAAAGGGGATGTTGGA	TCGCTCACTTCTTCTGAGAGC
Caspase-9	ATCGAGGATATTCAGCAGGCA	CCTCGGGTCTCAAGGTCTGT

## Data Availability

The original contributions presented in this study are included in the article. Further inquiries can be directed to the corresponding authors. Sequence data in this study were uploaded to the NCBI SRA database, and the SRA accession number is PRJNA1123850 (https://www.ncbi.nlm.nih.gov/bioproject/PRJNA1123850 (accessed on 14 January 2025).
